# Effect of Tb-doped Concentration Variation on the Electrical and Dielectric Properties of CaF_2_ Nanoparticles

**DOI:** 10.3390/nano8070532

**Published:** 2018-07-14

**Authors:** Xiaoyan Cui, Tingjing Hu, Jingshu Wang, Xin Zhong, Yinzhu Chen, Junkai Zhang, Xuefei Li, Jinghai Yang, Chunxiao Gao

**Affiliations:** 1 Key Laboratory of Functional Materials Physics and Chemistry of the Ministry of Education, National Demonstration Center for Experimental Physics Education, Jilin Normal University, Siping 136000, China; xycuimail@163.com (X.C.); jingshuwang126@126.com (J.W.); zhongxin@calypso.cn (X.Z.); yinzhuchen126@126.com (Y.C.); junkaizhang126@126.com (J.Z.); xuefeili163@163.com (X.L.); jhyang1@jlnu.edu.cn (J.Y.); 2 State Key Laboratory of Superhard Materials, Jilin University, Changchun 130012, China; chunxiaogao126@126.com

**Keywords:** nanoparticles, electrical properties, dielectric behavior, transport process

## Abstract

Calcium fluoride (CaF_2_) nanoparticles with various terbium (Tb) doping concentrations were investigated by X-ray diffraction (XRD), transmission electron microscopy (TEM), and alternating current (AC) impedance measurement. The original shape and structure of CaF_2_ nanoparticles were retained after doping. In all the samples, the dominant charge carriers were electrons, and the F^−^ ion transference number increased with increasing Tb concentration. The defects in the grain region considerably contributed to the electron transportation process. When the Tb concentration was less than 3%, the effect of the ionic radius variation dominated and led to the diffusion of the F^−^ ions and facilitated electron transportation. When the Tb concentration was greater than 3%, the increasing deformation potential scattering dominated, impeding F^−^ ion diffusion and electron transportation. The substitution of Ca^2+^ by Tb^3+^ enables the electron and ion hopping in CaF_2_ nanocrystals, resulting in increased permittivity.

## 1. Introduction

Lanthanide (III)-doped nanocrystals have attracted considerable attention due to their potential applications in electrical and optical devices [1,2,3,4,5]. Due to low refractivity, high transparency, and low phonon energy, fluoride compounds are well-known host materials for lanthanide (III)-doped nanocrystals [6,7,8,9,10,11]. As an important class of fluorides, calcium fluoride (CaF_2_) has been used as a host due to its stability and non-hygroscopic behavior. Therefore, lanthanide (III)-doped CaF_2_ nanoparticles have been examined in fundamental and applied studies [12,13,14,15,16,17].

For optical and optoelectronic devices, energy consumption is a key factor in evaluating their performance [18,19,20,21]. Energy consumption is inextricably tied to the electrical and dielectric performance of the material used in a device. Therefore, the electrical and dielectric properties of lanthanide (III)-doped CaF_2_ nanoparticles are worth exploring. Due to the presence of numerous grain boundaries, nanocrystals have many unique properties that would not be present in their corresponding bulk counterparts [22,23]. Additionally, electrical and dielectric properties are closely related to the charge carrier types and their scattering processes. However, the above subjects have not been studied in detail. 

In this study, the morphology and structure of CaF_2_ nanoparticles with various Tb doping concentrations are studied using X-ray diffraction (XRD) and transmission electron microscopy (TEM). The electrical and dielectric properties are investigated using alternating current (AC) impedance measurements. The transportation properties of charge carriers are also discussed.

## 2. Materials and Methods 

A series of Tb-doped CaF_2_ nanoparticles were synthesized using the liquid-solid-solution (LSS) solvothermal route [24,25,26]. The sample was synthesized as follows: 16.8 mL oleic acid, 48 mL ethanol, and 0.4 g sodium hydroxide (Sinopharm Chemical Reagent Beijing Co., Ltd, Beijing, China) were mixed together and stirred for 10 min; 1.888 g Ca(NO_3_)_2_·H_2_O (Sinopharm Chemical Reagent Beijing Co., Ltd, Beijing, China) dissolved in 20 mL H_2_O was added to the solution and stirred for 10 min. Then, 0.672 g sodium fluoride (NaF) (Sinopharm Chemical Reagent Beijing Co., Ltd, Beijing, China) dissolved in 20 mL H_2_O was added to the solution and stirred for 1 h. Finally, the solution was poured into an autoclave. The system was kept at 160 °C for 24 h and then cooled naturally in air. The product was centrifuged with cyclohexane (Sinopharm Chemical Reagent Beijing Co., Ltd, Beijing, China) and ethanol and dried at 80 °C. To the Tb-doped samples, part of the Ca(NO_3_)_2_·H_2_O was substituted by Tb(NO_3_)_3_·5H_2_O (Sinopharm Chemical Reagent Beijing Co., Ltd, Beijing, China) and the Tb(NO_3_)_3_·5H_2_O molar fractions were 1, 2, 3, 4, and 5 mol %. Transmission electron micrographs were measured by TEM (JEOL Ltd., Tokyo, Japan). The samples structure and phase were measured by XRD (Rigaku, Tokyo, Japan) with Cu Kα radiation (λ = 1.5406 Å). The sample we synthesized was powered, which is incompact. However, to complete impedance measurements, the sample must be compact. Therefore, the sample was pressed into a cylinder (ø6 × 1 mm) using a shock pressure (20 MPa). The impedance measurement was measured by parallel plate electrode at atmospheric pressure. The input voltage amplitude was 1 V, and frequency ranged from 0.1 to 10^7^ Hz. The output signal was gathered and processed by the impedance analyzer (Solartron 1260, Solartron, Hampshire, UK) with a dielectric interface (Solartron 1260, Solartron, Hampshire, UK).

## 3. Results and Discussion

**Figure 1:**
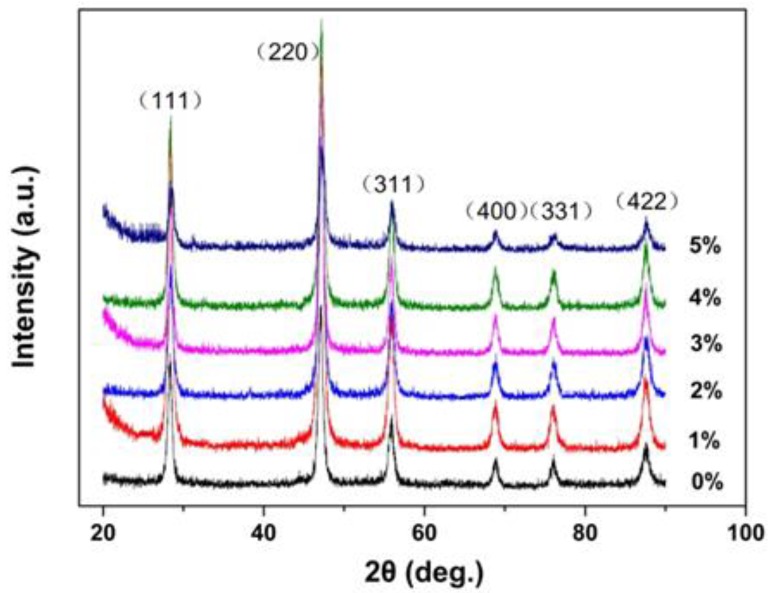
X-ray diffraction (XRD) patterns of terbium (Tb)-doped CaF_2_ nanocrystals.

**Figure 2:**
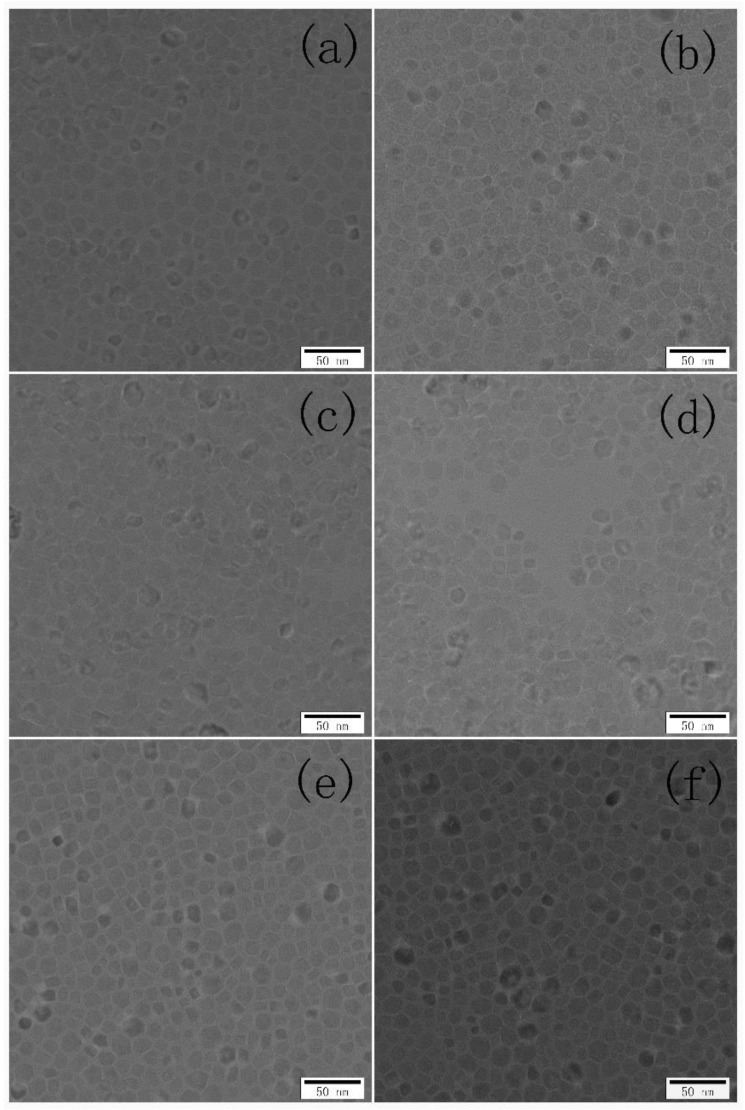
Transmission electron microscopy (TEM) image of the CaF_2_ nanoparticles with various Tb concentrations: (**a**) un-doped; (**b**) 1% Tb; (**c**) 2% Tb; (**d**) 3% Tb; (**e**) 4% Tb; and (**f**) 5% Tb.

**Figure 3:**
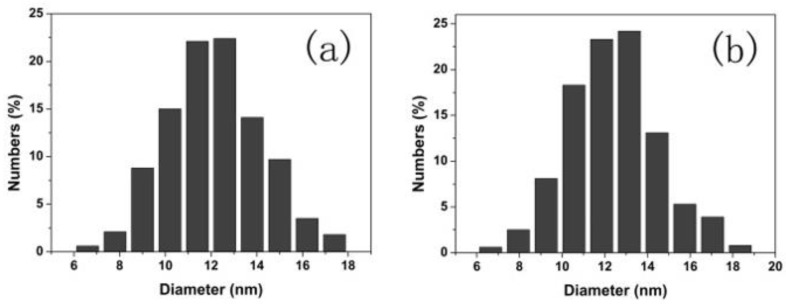

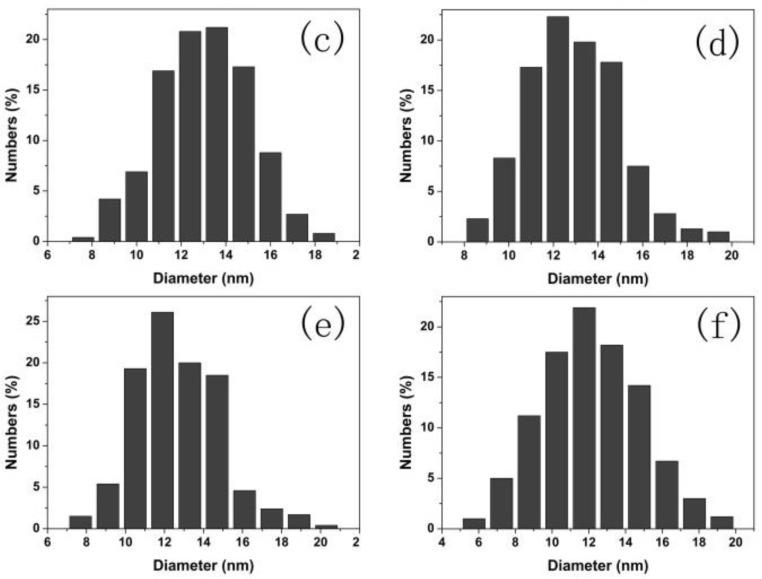
Size distribution histogram of the CaF_2_ nanoparticles with various Tb concentrations: (**a**) un-doped; (**b**) 1% Tb; (**c**) 2% Tb; (**d**) 3% Tb; (**e**) 4% Tb; and (**f**) 5% Tb.

**Figure 4:**
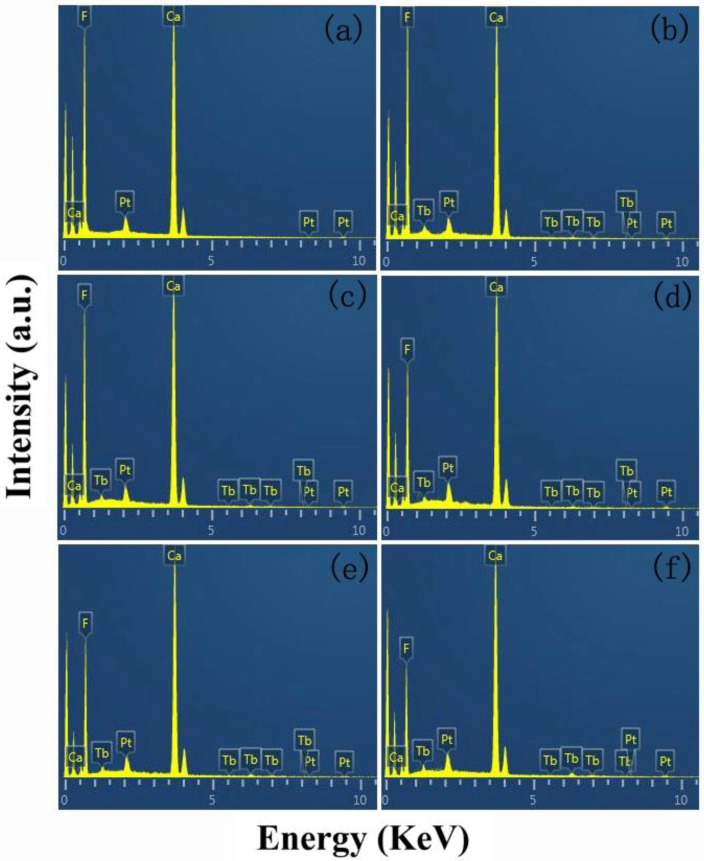
EDS spectrum of the CaF_2_ nanoparticles with various Tb concentrations: (**a**) un-doped; (**b**) 1% Tb; (**c**) 2% Tb; (**d**) 3% Tb; (**e**) 4% Tb; and (**f**) 5% Tb.

**Figure 5:**
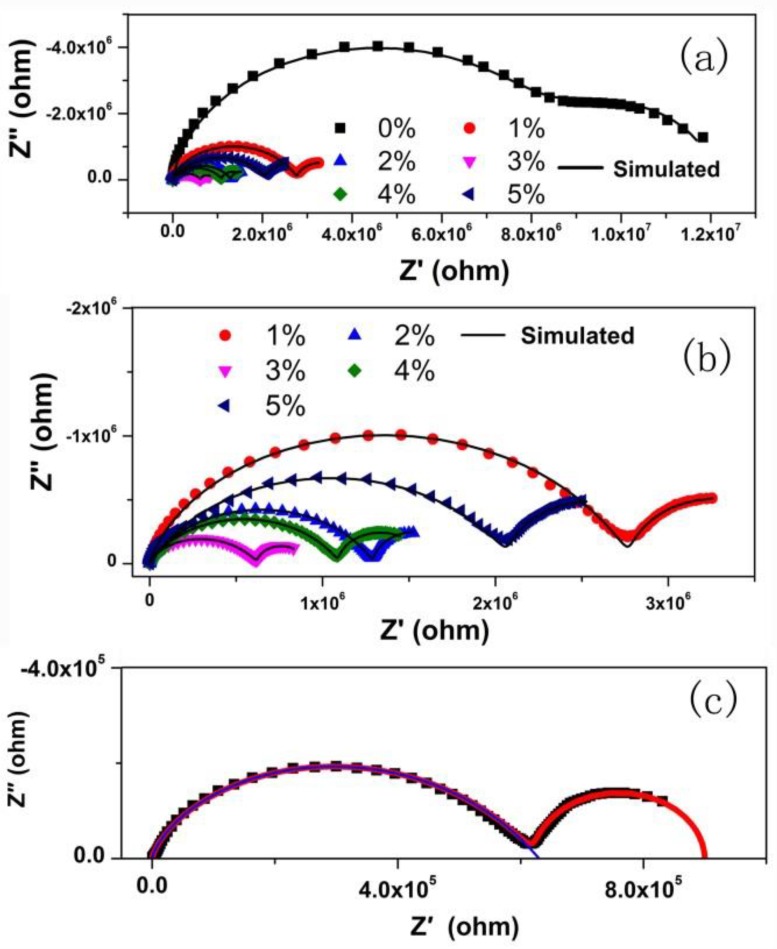
(**a**) The impedance spectroscopy of Tb-doped CaF_2_ nanocrystals; (**b**) The enlarged spectra with Tb concentrations ranging from 1% to 5%; (**c**) The spectroscopy with 3% Tb, where *R*_1_ and *R*_2_ are the *X*-axis intercepts of the spectroscopy.

Figure 1 shows the XRD patterns of the Tb-doped CaF_2_ nanoparticles. The peaks of all the samples matched well with the standard cubic CaF_2_ phase (JCPDS Card No. 35-0816), and no impurity phase was found in the spectra. Therefore, the crystal structure remained unchanged after doping. Figure 2, Figure 3 and Figure 4 shows the TEM image, size distribution histogram, and energy dispersive spectrometer (EDS) of the CaF_2_ nanoparticles with various Tb concentrations. We observed that all samples were square and the mean dimensions were all about 12 ± 3 nm. The presence of Tb in the EDS spectrums of Tb-doped CaF_2_ nanoparticles indicates that Tb was successfully doped into the samples. The impedance spectroscopy of CaF_2_ nanocrystals with various Tb concentrations is shown in Figure 5.

**Figure 6:**
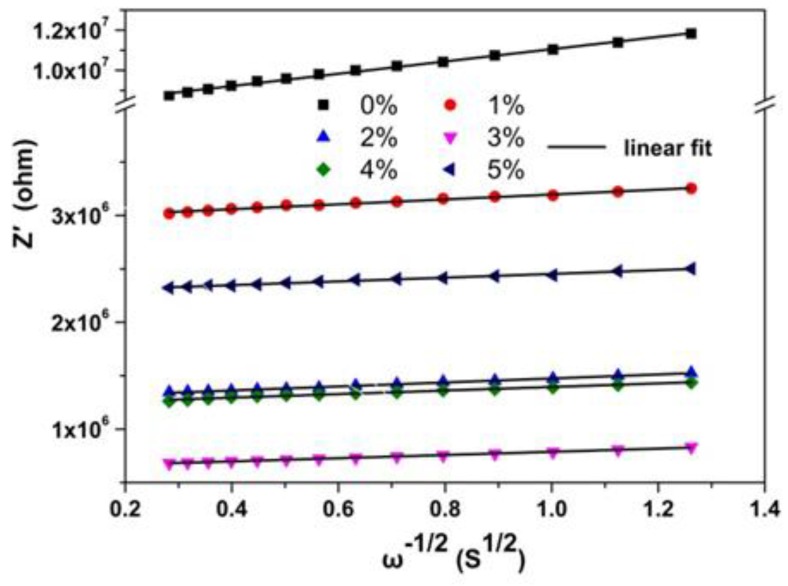
*Z′*~*ω*^−1/2^ of Tb-doped CaF_2_ nanocrystals in the low frequency region.

To quantify the effect of Tb doping on the electrical transport properties of CaF_2_ nanocrystals, an equivalent circuit was used to fit the impedance results. The alternative representation *Z′*~*ω*^−1/2^ was used to study the F^−^ ion transport property and the result is presented in Figure 6.

**Figure 7:**
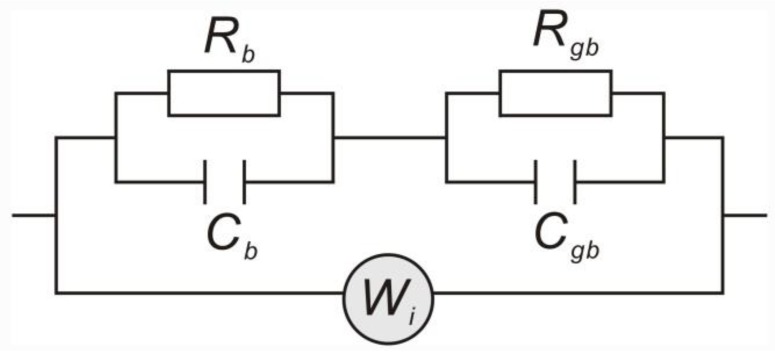
The equivalent circuit used to fit the impedance results. *R_b_* is grain resistance, *R_gb_* is grain boundary resistance, *C_b_* is grain capacitance, *C_gb_* is grain boundary capacitance, and *W_i_* is the Warburg impedance.

In the low frequency region, the *Z′* and *ω*^−1/2^ were linear, indicating the existence of F^−^ ion diffusion at low frequency. Thus, a Warburg element was used to depict the F^−^ ion conduction, which was added to the equivalent circuit diagram as presented in Figure 7. The fitted spectra agreed well with the experiment results (Figure 5), indicating that electron and ion conduction coexisted in the sample transport process.

**Figure 8:**
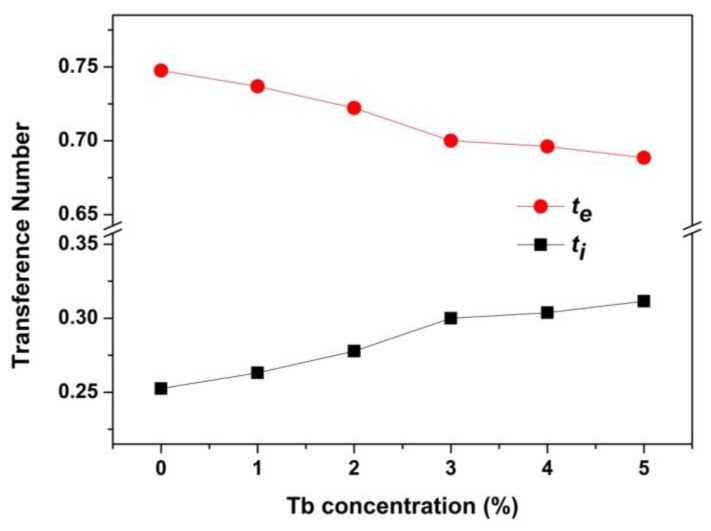
Tb concentration dependence of the ion transference number (*t_i_*) and the electron transference number (*t_e_*).

Considering the charge carriers include both ions and electrons, the transference number was used to describe the contribution of the ions and electrons to the transportation process [27]. The F^−^ ion transference number was defined as *t_i_* and electron as *t_e_*, so *t_i_* and *t_e_* can be expressed as:

(1) $$t_{i}=\left(R_{2}-R_{1}\right)/R_{2},$$

(2) $$t_{e}=R_{1}/R_{2},$$

where *R*_1_ and *R*_2_ are the *X*-axis intercepts of the spectroscopy (Figure 5c). The *t_i_* and *t_e_* of CaF_2_ nanocrystals with various Tb concentrations are presented in Figure 8. In all samples, the electron transport dominated, and the F^−^ ion transference number increased with increasing Tb concentration.

**Figure 9:**
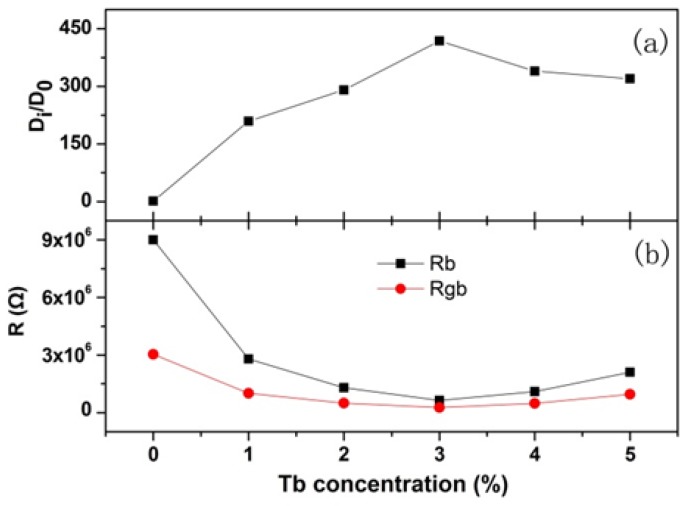
Tb concentration dependence of the diffusion coefficient (**a**), grain and grain boundary resistance (**b**). *D*_0_ represents the diffusion coefficient of un-doped CaF_2_ nanocrystals.

The Warburg coefficient (*σ*) can be obtained by the following equation [28]:

(3) $$Z^{\prime }=Z_{0}^{\prime }+\sigma \omega^{-1/2},$$

where $Z_{0}^{\prime }$ is a constant and *ω* is the frequency. By performing a linear fit on the *Z′*~*ω*^−1/2^ scatterplot (Figure 6), the Warburg coefficient of CaF_2_ nanocrystals with different Tb concentrations was obtained. The ion diffusion coefficient can be expressed as:

(4) $$D_{i}=0.5{\left(\frac{RT}{AF^{2}\sigma C}\right)}^{2},$$

where *R* is the ideal gas constant, *T* is temperature, *F* is the Faraday constant, and *C* is the molar concentration of F^−^ ions. The F^−^ ion diffusion coefficient for un-doped CaF_2_ nanocrystals was set as *D*_0_, and the *D_i_/D*_0_ of various Tb concentrations was obtained, as shown in Figure 9a. Through fitting the impedance spectra by the equivalent circuit, the grain and grain boundary resistances were obtained as shown in Figure 9b.

When the Tb concentration was less than 3%, the F^−^ ion diffusion coefficient increased with increasing Tb concentration; when the Tb concentration was greater than 3%, the F^−^ ion diffusion coefficient decreased. The grain and grain boundary resistances decreased with increasing Tb concentration until 3%, and then increased. In all samples, the grain resistance dominated in the total resistance, indicating that the defects in the grain region considerably contribute to the electron transportation process.

The changing of the transport properties with the replacement of Ca^2+^ by Tb^3+^ was analysed from two aspects: (1) the Tb^3+^ ionic radius being smaller than the Ca^2+^ ionic radius, which leads to the increasing mobility of the charge carriers [29]; and (2) due to the different valence, the replacement of Ca^2+^ by Tb^3+^ results in the deformation of the lattice and an increase in the deformation potential scattering, which decreases the mobility of the charge carriers. When the Tb concentration was less than 3%, the effect of ionic radius variation dominated, facilitating both F^−^ ion diffusion and electron transportation. However, when the Tb concentration was greater than 3%, the increasing deformation potential scattering was dominant, impeding F^−^ ion diffusion and electron transportation. 

**Figure 10:**
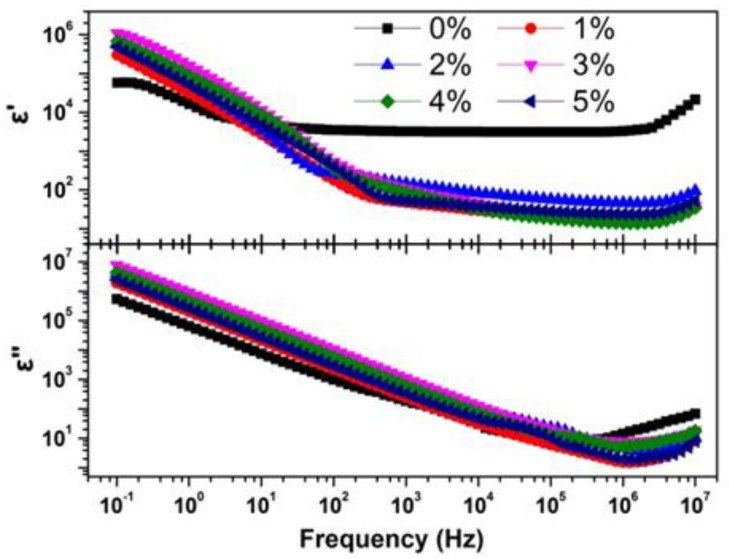
The complex permittivity (*ε*′ and *ε*″) vs. the frequency of CaF_2_ nanocrystals (*f*) with various Tb concentrations.

To comprehensively understand the transport properties of Tb-doped CaF_2_ nanoparticles, the dielectric properties were further studied. The complex permittivity (*ε*′, *ε*″) with frequency (*f*) of CaF_2_ nanocrystals under different Tb concentrations are shown in Figure 10.

The *ε*′ decreased linearly with increasing frequency in the low frequency region, then remained almost unchanged in the middle frequency region, finally increasing in the high frequency region. The *ε*″ decreased linearly with increasing frequency and then increased in the high frequency region. The presence of strong low-frequency dispersion in the permittivity implies that the electron and ion are hopping in the transport process [30]. At low frequencies, the *ε*′ and *ε*″ of Tb-doped samples were greater than of the un-doped sample, indicating Tb-doping facilitates electron and ion hopping in CaF_2_ nanocrystals. The substitution of Ca^2+^ by Tb^3+^ implies the creation of vacancy. Once a vacancy is created, further atom motion is relatively easy, so a neighboring atom hops into the vacancy, which is easily translated to another site, and finally facilitates charge carriers hopping and increases permittivity.

## 4. Conclusions

CaF_2_ nanoparticles with various Tb doping concentrations were characterized by XRD, TEM, and AC impedance. In all samples, the dominant charge carriers were electrons, and the F^−^ ion transference number increased with increasing Tb concentration. The defects in the grain region considerably contributed to the electron transportation process. When the Tb concentration was less than 3%, the ionic radius variation effect dominated and facilitated F^−^ ion diffusion and electron transportation. When the Tb concentration was greater than 3%, the increasing deformation potential scattering dominated, impeding F^−^ ion diffusion and electron transportation. The substitution of Ca^2+^ by Tb^3+^ enabled electron and ion hopping in CaF_2_ nanocrystals, and finally led to the increasing permittivity. We concluded that rare-earth-doping treatment is an effective method for modulating the electric conductive and dielectric performance of CaF_2_ nanoparticles. We expect that the design of CaF_2_-based optical and optoelectronic devices could benefit from our investigation.
